# eIF4E Is an Important Determinant of Adhesion and Pseudohyphal Growth of the Yeast *S. cerevisiae*

**DOI:** 10.1371/journal.pone.0050773

**Published:** 2012-11-30

**Authors:** Daniela Ross, Manisha Saxena, Michael Altmann

**Affiliations:** Institute of Biochemistry and Molecular Medicine, University of Bern, Bern, Switzerland; The John Curtin School of Medical Research, Australia

## Abstract

eIF4E, the cytoplasmatic cap-binding protein, is required for efficient cap-dependent translation. We have studied the influence of mutations that alter the activity and/or expression level of eIF4E on haploid and diploid cells in the yeast *S. cerevisiae*. Temperature-sensitive eIF4E mutants with reduced levels of expression and reduced cap-binding affinity clearly show a loss in haploid adhesion and diploid pseudohyphenation upon starvation for nitrogen. Some of these mutations affect the interaction of the cap-structure of mRNAs with the cap-binding groove of eIF4E. The observed reduction in adhesive and pseudohyphenating properties is less evident for an eIF4E mutant that shows reduced interaction with p20 (an eIF4E-binding protein) or for a p20-knockout mutant. Loss of adhesive and pseudohyphenating properties was not only observed for eIF4E mutants but also for knockout mutants of components of eIF4F such as eIF4B and eIF4G1. We conclude from these experiments that mutations that affect components of the eIF4F-complex loose properties such as adhesion and pseudohyphal differentiation, most likely due to less effective translation of required mRNAs for such processes.

## Introduction

Eukaryotic translation is initiated by the interaction of the 5′ end of mRNAs with eIF4F, a complex of proteins formed by eIF4E, the cap-binding protein, eIF4G, a scaffold protein and eIF4A, a helicase which helps to unwind secondary structures of mRNAs. In higher cells, the interaction of eIF4E with eIF4G is regulated by eIF4E-BPs, small acidic proteins which impede their interaction by binding to eIF4E. When translation takes place, eIF4E-BPs become hyperphosphorylated by the kinase Tor1 dissociating thereby from eIF4E and allowing for the formation of the eIF4F complex. Overexpression of eIF4E in mammalian cells is an important determinant of cell proliferation which is observed in several cancer forms [1]. Accordingly, different strategies are now under clinical trial to downregulate the activity or concentration of eIF4E to impede cell growth [2], [3].

In the unicellular yeast *S. cerevisiae*, eIF4E is an essential component of protein synthesis. Several mutants of eIF4E with reduced cap-binding activity have been obtained which render a temperature-sensitive phenotype and arrest cell growth at non-permissive temperatures [4], [5]. At least two eIF4E-BPs, called p20 and Eap1 exist in *S. cerevisiae* which interact with eIF4E and compete thereby for its interaction with eIF4G [6], [7]. Previous studies have shown, that diploid yeast cells carrying a knockout of the non-essential genes encoding p20 (caf20) and Eap1 loose their tendency to form pseudohyphae [8], [9]. Pseudohyphenation of diploid yeast cells is due to reprogramming observed when cells are exposed to nutritional limitations such as low nitrogen concentrations. This developmental switch is under the control of downstream effectors of the cAMP/PKA, Snf1 and MAPK pathways and allows the cells to forage the environment for better nutritional conditions [10]. More recently, the importance of the signal transduction pathway which regulates Tor1-activity has been described as a further determinant of the developmental switch which leads to pseudohyphenation upon nitrogen starvation (for a recent review, see [11]).

**Figure 1:**
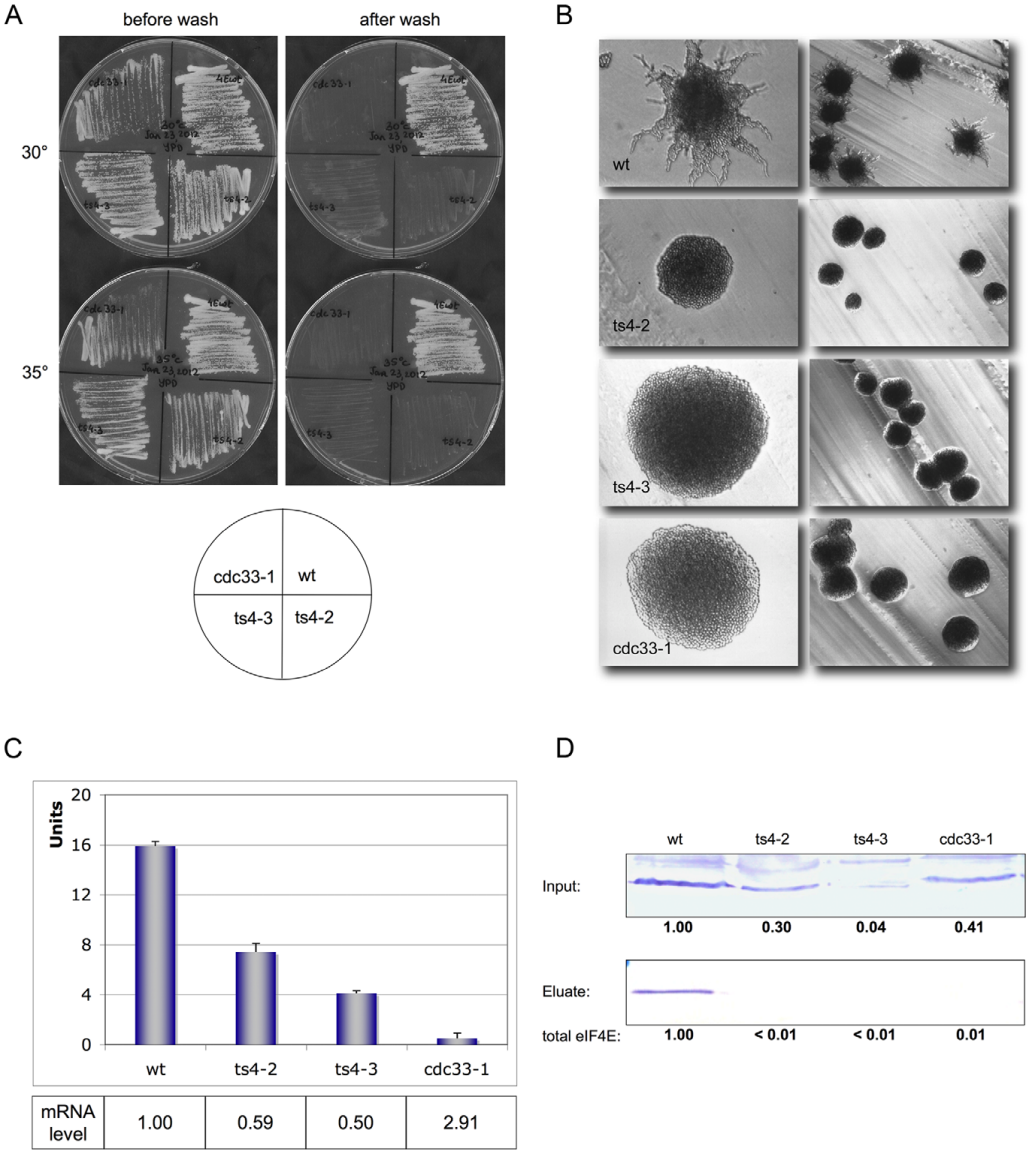
eIF4E temperature-sensitive mutants loose adhesion, pseudohyphenation and cap-interaction. (**A**) Adhesion of haploid eIF4E mutants ts4-2, ts4-3, cdc33-1 in comparison to eIF4E wt. Plates were incubated at 30° or 35°C for 2 days, then washed under a gentle stream of water. (**B**) Pseudohyphenation of diploid eIF4E ts mutants in comparison to eIF4E wt. Cells were incubated on SLAD_50_ (50 µM ammonium sulphate) plates at 30°C for 2 days; shown is a 200× or 40× magnification of cells. (**C**) ß-Galactosidase activity expressed from *Flo11-LacZ* in haploid eIF4E wt and mutants ts4-2, ts4-3 and cdc33-1. Expression levels were normalized to LacZ mRNA content which was determined by quantitative RT-PCR. (**D**) Western Blots of eIF4E ts mutants. *Top panel:* Blot of total extracts used for incubation with m^7^GDP-agarose (^1^/_20_ volume input; 50 µg total protein); *lower panel:* Blot of eluted eIF4E (^1^/_1_ volume). Intensity of eIF4E signals was analysed by ImageJ. Protein inputs for the upper blot were normalized with the help of a polyclonal antibody against carboxypeptidase Y (Prc1p; not shown), numbers represent the relative eIF4E content as compared to wt protein.

Haploid yeast cells do not form pseudohyphae but can adhere to organic or anorganic surfaces and penetrate thereby other cells. Such a condition which was previously known for pathogenic yeasts species such as *C. albicans* or *C. glabrata* has been also observed in recent years for *S. cerevisiae* strains which cause severe problems to patients with reduced immunoresistance [12].

For both adhesion and pseudohyphenation, expression of the cell adhesion protein Flo11 is an important determinant. The promoter region of the gene encoding Flo11 is regulated by transcription factors such as Flo8, which is not expressed in non-filamentous yeast strains and as Gcn4, which is induced upon amino acid starvation. Several signal transduction pathways converge and regulate the level of Flo11-mRNA expression (reviewed in [11]).

It has been reported that inhibition of protein synthesis plays a role for the commitment of yeast cells to enter differentiation programs that lead to adhesion and pseudohyphenation. It is not clear if those inhibitory effects are due to inhibition of global protein synthesis or inhibition of particular mRNAs. So, rapamycin which inhibits the TOR protein kinases leads to inhibition of pseudohyphenation of diploids but not to loss of adhesion of haploids [13]. Inhibition of adhesive properties has been also observed for cells treated with cycloheximide which inhibits elongation of translation [13]. Further studies in yeast indicate that the lack of Rack1, a ribosome associated protein or of one copy of ribosomal protein rps26 abolishes the expression of Flo11 and leads to inhibition of filamentation and adhesive growth [14], [15]. Additionally, a mechanism which allows for cap-independent translation of mRNAs such as transcription factor Flo8 and other proteins involved in adhesion and filamentation has been proposed [16]. Cap-independent translation is suppossed to occur in yeast cells under conditions of stress or nutritional deprivation. Under such conditions, eIF4E-activity is reduced by sequestration into stress granules [17] or is completely abolished when cells are maintained in the stationary phase of growth [18].

We report here, that cap-dependent translation is an important determinant of adhesive growth and pseudohyphenation as haploid and diploid yeast strains carrying mutations in eIF4E as well as knockouts of components of the eIF4F-complex such as eIF4G1 or eIF4B loose these properties.

## Materials and Methods

### Yeast Strains, Plasmids and Media

*S. cerevisiae* strains used in this study are listed in Table S2, plasmids in Table S3. Deletion of eIF4E or p20 was obtained by directly transforming PCR products obtained from amplification of eIF4E::KanX or p20::NatR into competent RH2585 (a generous gift of G. Braus, Georg-August-Universität Göttingen, Germany). Because eIF4E is an essential protein, survival was maintained by an eIF4E gene copy on a pVT-URA3 plasmid. Plasmids were amplified and isolated from *E. coli* strain XL2blue. Site-directed mutagenesis to produce the required mutation in the open-reading frame of eIF4E was performed on pCEN16-eIF4E plasmid (oligonucleotide pairs are listed in Table S4; [19]). Plasmids with mutated forms were transformed into RH2585 ΔeIF4E::KanX <pVTU-eIF4E> and cells were selected on synthetic media (SD: 0.67% Yeast Nitrogen Base, 2% Dextrose, 2% agar, 20 µg/mL Histidine). Plasmids were shuffled by using 5-FOA (fluoro oroctic acid) and selecting for the loss due to segregation of *URA3* plasmids [20]. Diploid mutant eIF4E strains were obtained by crossing haploid cell lines to the opposite mating type RH2586 ΔeIF4E::KanX <pVT-URA3 eIF4E> and positively selecting for *ura3^-^* clones on 5-FOA.

**Figure 2:**
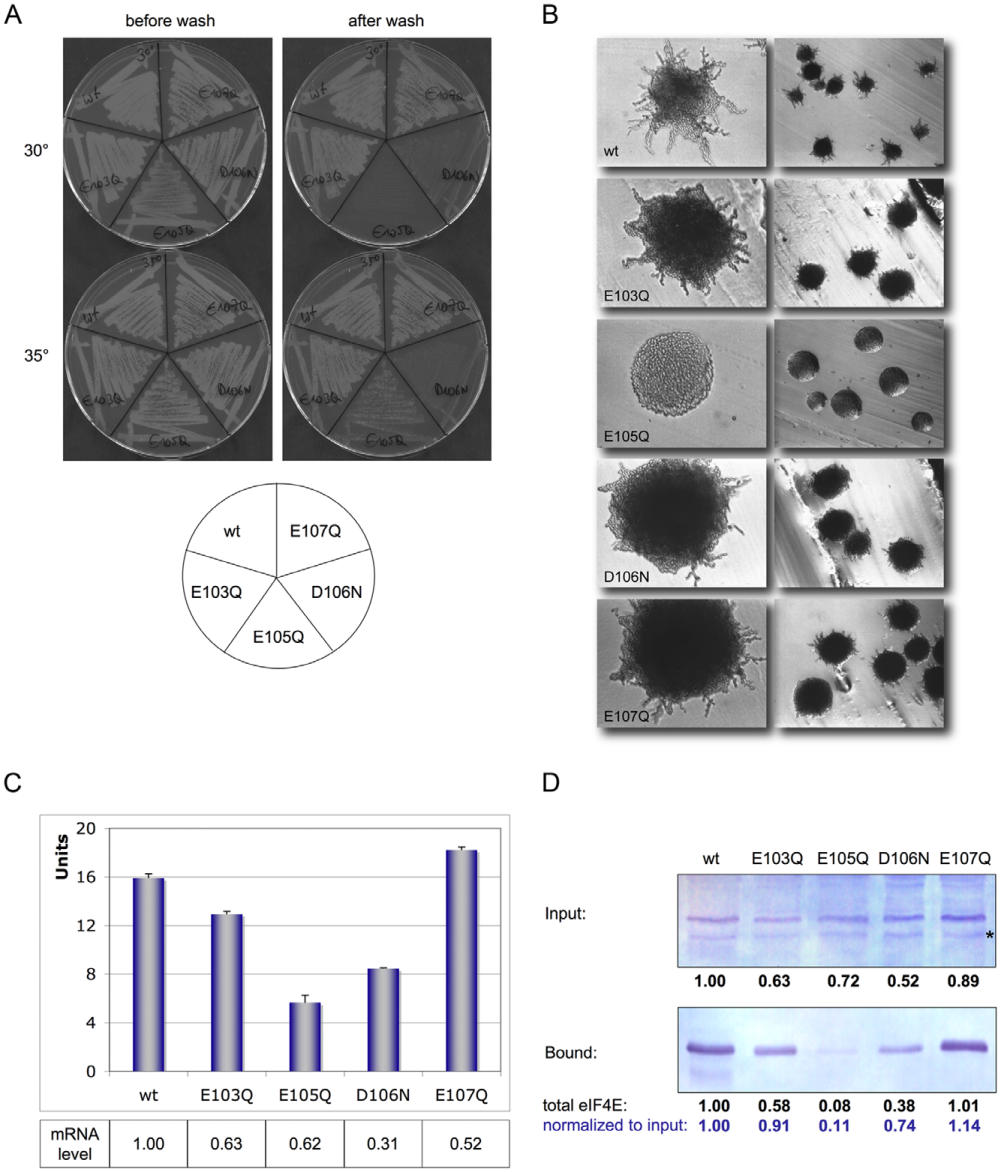
eIF4E mutations with reduced cap-interaction lead to loss of adhesion and pseudohyphenation. (**A**) Adhesion of haploid eIF4E cap-binding mutants E103Q, E105Q, D106N and E107Q in comparison to eIF4E wt. Plates were incubated at 30° or 35°C for 2 days, then washed under a gentle stream of water. (**B**) Pseudohyphenation of diploid eIF4E cap-binding mutants in comparison to eIF4E wt. Cells were incubated on SLAD_50_ plates at 30°C for 2 days; shown is a 200× or 40× magnification of cells. (**C**) ß-Galactosidase activity expressed from *Flo11-LacZ* in haploid eIF4E wt and mutants E103Q, E105Q, D106N and E107Q. Expression levels were normalized to LacZ mRNA content which was determined by quantitative RT-PCR. (**D**) Western Blot of eIF4E mutants. Blot of total extracts used for incubation with m^7^GDP-agarose (^1^/_20_ volume input; 50 µg total protein); *lower panel:* Blot of eluted eIF4E (^1^/_1_ volume). Intensity of eIF4E signals was analysed by ImageJ. Protein inputs for the upper blot were normalized with the help of a polyclonal antibody against carboxypeptidase Y (Prc1p; not shown), numbers represent the relative eIF4E content as compared to wt protein. Eluted eIF4E bands were furthermore normalized against total eIF4E input as determined for each extract (in blue). Asterix indicates an unspecific band.

### Phenotype Investigation

To test for adhesion, haploid cells were streaked out on YPD plates, incubated for 2 days at 30° or 35°C and washed with a gentle stream of water. Pseudohyphenation was tested on nitrogen limited SLAD_50_ plates (50 µM ammonium sulphate, 0.17% Yeast Nitrogen Base without ammonium sulfate, 2% Dextrose, 2% agar, 5 µg/mL uracil and histidine). Cells were streaked out and incubated for 3 days at 30°C.

### SDS-PAGE and Immunoblotting

Overnight cultures of haploid yeast mutant strains were harvested, 5 * 10^6^ cells (corresponding to ½ OD_600_) were pelleted, boiled in 2× SDS sample solution and loaded onto freshly prepared 17.5% SDS-PAGE gels [21]. After gel electrophoresis proteins were transferred to nitrocellulose membranes (BioRad, California) by Western blotting [22]. Blots were decorated with (1∶1000 dilution in 0.5% BSA, TBS) polyclonal rat antibodies against eIF4E or p20 and subsequently treated with polyclonal rabbit anti-rat IgG-HRP (1∶3000 dilution in 0.5% BSA, TBS) (Dako, Denmark). Blots were stained with freshly prepared 180 ppm chloro-naphtol and 40 ppm H_2_O_2_ in TBS (10 mM Tris.HCl pH 7.5, 150 mM NaCl). Intensity of stained proteins was analysed by ImageJ (Rasband, 1997–2012) and compared to wild type eIF4E signals.

### Binding Assay on m^7^GDP Agarose Resin

Overnight cultures of haploid yeast mutant strains were grown at 30°C to an OD_600_ of 1.0 to 2.0, harvested, washed with buffer ADP (30 mM Hepes-KOH pH 7.4, 100 mM KOAc, 2 mM Mg(OAc)_2_, 2 mM DTT, 0.1 mM PMSF) and resuspended in buffer ADP. Total cell extracts were obtained by treating cells with glass beads and protein concentration determined subsequently [23]. m^7^GDP agarose was washed twice with buffer ADP, previous to adding 0.7–1.0 mg of total protein extract and incubating at 4°C for 2 hours. Unbound protein was removed, resin washed three times with buffer ADP and either incubated for elution at 4°C in 1 mM m^7^GDP (in ADP buffer) for 15 minutes or protein bound to resin was directly applied onto SDS PAGE gels after boiling in 2× SDS sample solution.

**Figure 3:**
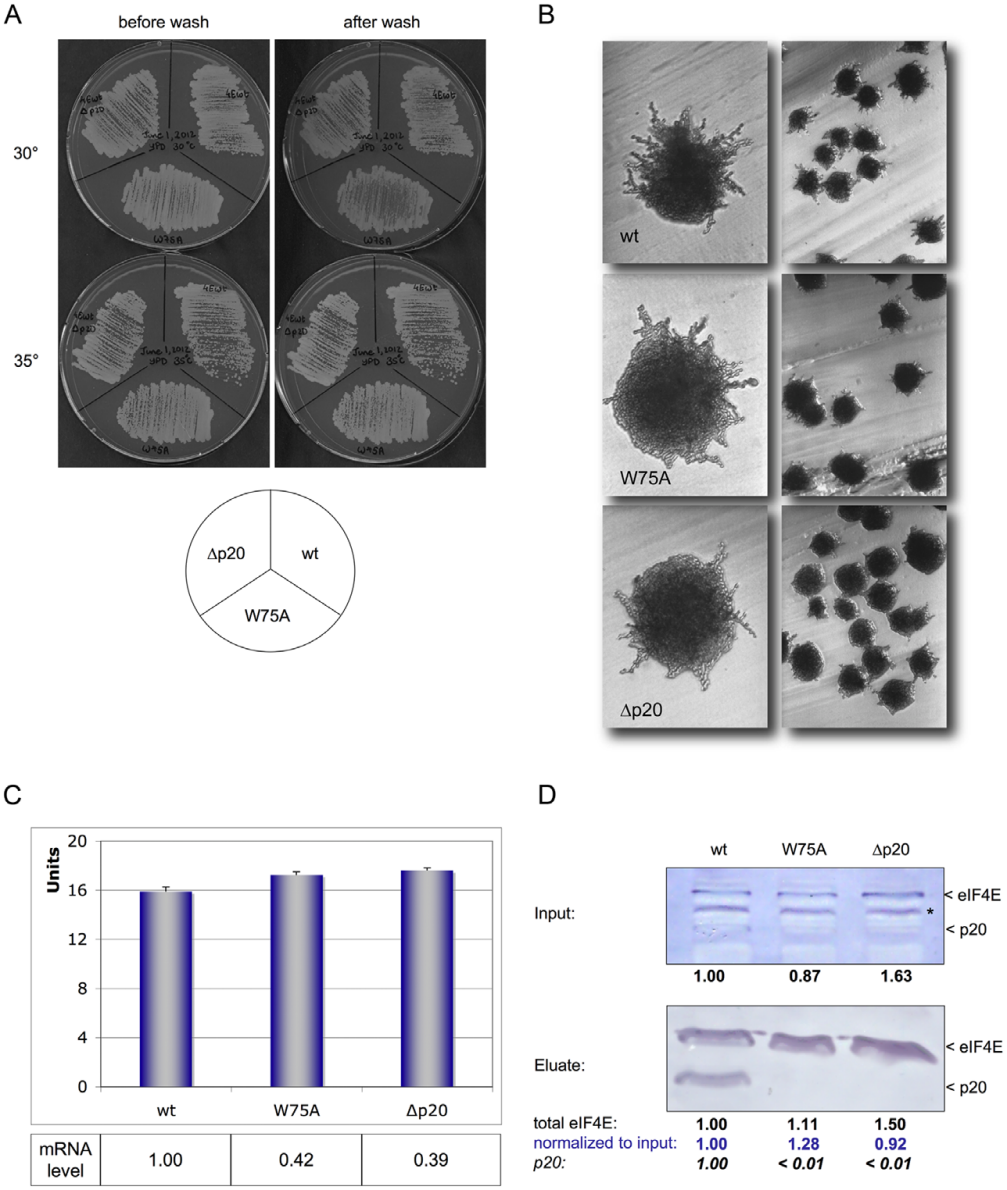
eIF4E mutants W75A (affecting p20 interaction) or a knockout of p20 do not loose adhesion and pseudohyphenation. (**A**) Adhesion of haploid eIF4E mutants W75A or Δp20 as compared to eIF4E wt. Plates were incubated at 30° or 35°C for 2 days, then washed under a gentle stream of water. (**B**) Pseudohyphenation of diploid eIF4E W75A or Δp20 in comparison to eIF4E wt. Cells were incubated on SLAD_50_ plates at 30°C for 2 days; shown is a 200× or 40× magnification of cells. (**C**) ß-Galactosidase activity expressed from *Flo11-LacZ* in haploid eIF4E wt and mutants W75A and Δp20. Expression levels were normalized to LacZ mRNA content which was determined by quantitative RT-PCR. (**D**) Western Blots of eIF4E wt, W75A or Δp20. *Top panel:* Blot of extract used for binding to m^7^GDP-Agarose (^1^/_20_ volume of input, 50 µg total protein each lane); *lower panel:* Blot of total eIF4E bound to m^7^GDP-Agarose (1 mg input), additional decoration with polyclonal antibody against p20. Intensity of eIF4E signals was analysed by ImageJ. Protein inputs for the upper blot were normalized with the help of a polyclonal antibody against carboxypeptidase Y (Prc1p; not shown), numbers represent the relative eIF4E content as compared to wt protein. Eluted eIF4E bands were furthermore normalized against total eIF4E input as determined for each extract (in blue). Asterix indicates an unspecific band. Signal strength of p20 is indicated in cursive numbers.

### LacZ-assay from Extracts

LacZ-assays of RH2585 ΔeIF4E::KanX <pCEN16-eIF4E wt/mutation> strains transformed with the plasmid yep355 *Flo11-LacZ (*promoter region and 5′UTR of Flo11 fused to the LacZ reporter gene; a generous gift of G. Fink, Whitehead Institute for Biomedical Research, MA*)* [24] were performed. Total cell extracts were obtained by treating cells resuspended in 0.1 M Tris-HCl pH 8.0, 10% Glycerol, 1 mM DTT with chilled glass beads and protein concentrations were determined [23]. LacZ-assays were performed at 28°C with o-nitrophenyl-ß-D-galactoside (ONPG) as substrate; 1 ß-Galactosidase Unit corresponds to 1 nmol hydrolysed ONPG per minute and mg protein.

### Quantitative RT-PCR

Total RNA isolation from yeast cells (transformed with the plasmid *Flo11-LacZ*) was done according to a slightly modified phenol:chloroform extraction protocol as described in the Molecular Cloning Laboratory Manual by Sambrook, Fritsch and Maniatis (1990). To eliminate genomic DNA contamination, DNase I treatment was performed (Roche, No. 04 716 728 001) and RNA concentrations determined (A_260_/A_280_). RNA was reverse-transcribed using MultiScribe™ Reverse Transcriptase and Random Hexamers (Applied Biosystems). Oligonucleotides for quantification of LacZ expression (or Act1 and Fba1 as stable reference genes) were designed to amplify PCR products of 70 to 150 bp [25]. For best specificity of oligonucleotides, a BLAST analysis of the *S. cerevisiae* genome was performed as well as an analysis to avoid secondary structures or self- and cross-dimers using Primer Express 3.0 (Applied Biosystems). The complete set of oligonucleotides used in this study is listed in table S4. Real-time PCR was performed in MicroAmp® optical 384-well reaction plates (10 µL reaction volume). Fast SYBR® Green Master Mix was mixed with oligonucleotide pairs (0.9 µM final concentration) and 10, 2.5 or 0.625 ng cDNA were used per well. Assays were conducted in triplicates and a non-template-control was also incorporated for each oligonucleotide pair. Samples were analyzed via the 2ΔΔCt method [26] with an Applied Biosystem ViiA™ 7 PCR machine and melting-curve data were collected. ß-Galactosidase activity Units were normalized according to determined *Flo11-LacZ* mRNA levels.

## Results

### Temperature-sensitive eIF4E Yeast Mutants Loose Adhesion and do not Pseudohyphenate

Using plasmid shuffling techniques (see Table S3; Material and Methods) we introduced eIF4E-mutations ts4-2 (G179D/E73K), ts4-3 (G179D/E103K) and cdc33-1 (G113D) into the adhesive haploid yeast strain RH2585 (see Table S2). They all render a temperature-sensitive phenotype (no growth at 37°C; see Figure S1) [4]. As shown in Figure 1A, ts-strains grown for 2–3 days on full medium at two different temperatures (they still grow at 35°C, though rather slowly) almost completely lost adhesion when compared to the isogenic strain carrying wt (wild type) eIF4E. We confirmed the presence of eIF4E protein by SDS-PAGE and Western Blotting. For ts-mutants 4–2 and 4–3, but not for cdc33-1 much lower eIF4E-levels were detected when compared to wt (Figure 1D). This was surprising, as these mutants grew quite well at temperatures between 20°C and 30°C and even though eIF4E is an essential protein in yeast [27]. We assume, that at least under these conditions eIF4E is not limiting.

To study the properties of mutant proteins, extracts were prepared from the different cell lines grown at 30°C and incubated with ^7^mGDP-resin. Only in the case of wild type cells, eIF4E was detected in the ^7^mGDP eluate (Figure 1D). We also purified extracts from strains 4–2 and 4–3 grown at 20°C (they express 1.5- to 2-fold as much eIF4E as when grown at 30°C) with ^7^mGDP-resin and couldn’t detect significant binding (not shown). We conclude that both, the loss of eIF4E-quantity and cap-binding activity of ts mutants are responsible for the loss of adhesion to a solid surface. We extended our studies by producing diploid cell lines which express wt or mutant eIF4E. Diploid temperature-sensitive cell lines completely loose the ability to form pseudohyphae when incubated under limiting nitrogen conditions (see Figure 1B).

In order to quantify the observed effects, we used a Flo11-LacZ plasmid construct as a reporter to expression levels. The level of Flo11-expression (LacZ Units; normalized to Flo11 mRNA levels) was considerable lower for ts mutants 4–2 and 4–3 and at background level for ts-mutant cdc33-1 when compared to wt cells (see Figure 1C). When determing mRNA-levels by quantitative PCR, we reproducibly observe an significantly increased Flo11 mRNA level for mutant cdc33-1 (almost 3× higher than wt) which does not compensate for low Flo11-expression (Fig. 1C).

All diploid cells (including those expressing wild type eIF4E) showed a very low level of Flo11-expression when grown in minimal SD medium supplemented with essential amino acids which hardly allowed for a proper correlation with pseudohyphenating properties. Only when diploid cells were incubated in SLAD medium with limiting ammonium concentrations (50 µM; about 1000× lower concentration of ammonium salt than normal SD medium), activation of Flo11-LacZ expression could be observed for diploid cells carrying wt eIF4E but not for diploid cells carrying eIF4E ts-mutants (not shown).

### eIF4E Mutants in the Cap-binding Slot Loose Adhesion

As ts-mutants which showed both, low expression and defects in binding to cap-analogs, had lost adhesive and pseudohyphenating properties, we decided to investigate the effect of 4E-mutations in the cap-binding groove. Especially interesting seemed mutations in acidic residues close to tryptophane W104 on eIF4E (which is known to interact by stacking with the purine ring of ^7^mG-capped mRNAs) such as E103Q (glutamate 103 to glutamine), E105Q (glutamate 105 to glutamine), D106N (aspartate 106 to asparagine) and E107Q (glutamate 107 to glutamine; see also Figure S2). Most notably, the negative charge of residue E105 has been described to interact electrostatically with the positive charge of m^7^G, stabilizing thereby the interaction of capped mRNA with eIF4E [28]. Not surprisingly, this mutant has a strong slow growth and ts-phenotype (Figure S1) which is not due to a loss in interaction of eIF4E with its partners p20 and/or eIF4G (see Table S1). The consequences of eIF4E mutation E105Q were evident in both haploid and diploid cells as adhesion and pseudohyphenation were completely lost (Figures 2A and 2B) which for haploid cells was also confirmed by a substantial decrease in Flo11-LacZ expression (Figure 2C). E105Q protein level was somewhat reduced as compared to wild type cells but almost none of mutant E105Q protein was bound to ^7^mGDP resin (Figure 2D).

Less pronounced was the effect of mutant D106N which is next to E105 but less proximal to the positively charged N6-imino group of ^7^mG (Figure S2). We did not detect notable growth defects for eIF4E mutant D106N (Figure S1) or loss of interaction with eIF4E partners (Table S1), haploid cells showed a pronounced loss of adhesivity but diploid cells only a mild loss of pseudohyphenation (Figure 2A and B). The loss in adhesivity of haploid mutant D106N is reflected in a reduction in Flo11-LacZ expression (Figure 2C). We probably detect gradual effects in adhesive properties when studying this mutant as binding of D106N protein to m^7^GDP was only partially reduced as compared to wt eIF4E (Figure 2D).

Less affected were mutants E103Q and E107Q which both showed comparable adhesion and pseudohyphenation levels as wt eIF4E (Figure 2A and B). Though also localised in close proximity to stacking tryptophane 104, they don’t contact the positively charged ^7^methylG imino-group (Figure S2). Expression of eIF4E mutant E103Q but not of E107Q was reduced when compared to wt. Mutated proteins bound as efficiently as wt eIF4E to ^7^mGDP resin (Figure 2D) and normalized Flo11-LacZ values were in the range of that of wild type cells (Figure 2C).

### An eIF4E Mutation Affecting Interaction with p20 does not Loose Adhesion

We also investigated if a mutation which affects eIF4E’s interaction with p20 would result in reduced adhesive properties. For this purpose we selected mutant W75A which is localised in the convex domain of eIF4E opposite to the cap-binding groove (see Figure S2) and which is known to be responsible for its interaction with other partners [29]. This mutant shows strongly reduced interaction with p20 and with eIF4G (see Table S1) and has a temperature-sensitive phenotype at 37°C (Figure S1). Surprisingly, W75A shows no loss of adhesion and only mild loss of pseudohyphenation when compared to wild type cells. (Figures 3A and B). Normalized Flo11-LacZ activity of W75A (Figure 3C), eIF4E protein level and binding to ^7^mGDP was comparable to wt eIF4E (Figure 3D). As expected, hardly any p20 was bound to eIF4E W75A (Figure 3D). We assume that temperature-sensitive mutant W75A is still capable – though at a reduced level - to interact with eIF4G and to perform cap-dependent translation to an extend that does not affect its adhering properties.

We wanted to confirm the significance of p20 for adhering properties of yeast strains previously described [8]. Deletion of p20 leads to a temperature-sensitive phenotype of adhesive strain RH2585 (see Figure S1). We only detect a mild loss in adhesion of haploid Δp20 or reduced pseudohyphenation of diploid Δp20 knockout strains when compared to diploid wild-type (Figures 3A and 3B). As well Flo11-LacZ levels (Figure 3C) as eIF4E level and binding to ^7^mGDP (Figure 3D) was not affected by the lack of p20.

We also investigated if knockout mutations in other initiation factors which are part of the eIF4F complex affect adhesive and pseudohyphenating properties of yeast cells. For this purpose, we analysed mutants carrying an individual knockout of tif1, tif2 (both encode eIF4A), tif3 (encodes eIF4B), tif4631 (encodes eIF4G1) or tif4632 (encodes eIF4G2). While individual deletion of each of both eIF4A-gene copies only had a very mild effect, deletion of eIF4B and eIF4G1 lead to a significant loss in adhesion and pseudohyphenating properties (Figure S3). These results clearly indicate that these properties are not only dependent on eIF4E-activity but also rely on other components of the eIF4F complex. Surprisingly, deletion of eIF4G2 had an opposite effect as we detected increased adhesive and pseudohyphenating properties of the knockout strain when compared to wt cells (Figure S3).

## Discussion

This study shows that mutations in eIF4E and knockouts of components of the eIF4F complex influence adhesive properties of haploid yeast cells and the ability of diploid cells to undergo pseudohyphenation upon nitrogen starvation.

Especially well studied here were mutants that affect eIF4E expression levels and activity. One of those mutations (E105Q) was localised in the cap-binding groove affecting its interaction with the cap structure of mRNAs. It is not known, if defects in this interaction affect the translation of all capped mRNAs to a similar extend or if the nucleotides following the cap further modulate this effect. A further electrostatic interaction which has been shown to stabilize interaction with capped mRNAs is due to positive charges on eIF4E interacting with the negative charges of the three phosphate residues which form the unusual link of ^7^mG to the second nucleotide at the 5′-end of capped mRNAs (which is often also a G) [28]. We have created eIF4E mutants K114L (lysine 104 to leucine), R157L (arginine 157 to leucine) and K162L (lysine 162 to leucine) abolishing nearby positive charges which could interact electrostatically with phosphate residues. All three basic residues (especially R157) are among the most conserved amino acids of eIF4E from different eukaryotic species [30]. None of these mutants were lethal, but especially R157L has a strong slow growth and temperature-sensitive phenotype. All 3 mutants showed reduced adhesion, especially haploid R157L which did not adhere and showed no pseudohyphenation (results not shown).

Surprisingly, eIF4E’s level and activity can be substantially reduced in yeast cells without having negative effects on growth under laboratory conditions as it is shown for our eIF4E temperature sensitive strains. Strong reductions in eIF4E level without major effects on overall translation have been recently shown for mammalian cells [31]. Nevertheless, our eIF4E ts-mutants have clearly lost adhesive and pseudohyphenation properties which might be of upmost importance for the survival of yeast strains in a natural environment characterized by sudden changes in temperature, humidity and nutritional conditions and where yeasts have to compete with many other organisms for survival.

A mutation in yeast eIF4E (W75A) which affects its interaction with p20 or a knockout strain of p20 do not show a notable decrease in these properties. This is opposed to previously published data describing loss of pseudohyphenation in a diploid homozygous Δp20 knockout strain [8]. We don’t have an explanation for these contradicting data. We conclude that the presence or absence of p20 is a less decisive factor for adhesive properties of yeast strains such as those examined in this work. This does not exclude that eIF4E-p20 interaction might modulate the translational rate of certain genes required for adhesive properties [32].

As shown in this paper, in the yeast *S. cerevisiae* cap-dependent translation plays an important role for adhesion (to solid phases) of haploids and helps to trigger the differential program for pseudohyphenation upon nutritional starvation of diploids. This seems to contradict previous reports indicating the importance of cap-independent translation for proper expression of proteins involved in such processes. As an explanation, we would like to propose that signalling induced by nutritional starvation and allowing for cap-independent translation [16] is required for such differentiation processes. Once that such programs have been triggered, cap-dependent translation will still be required to allow for proper expression of e.g. housekeeping genes. Inhibition of adhesion can also be observed when elongation of translation is partially inhibited by adding to the medium limiting concentrations of cycloheximide (20–50 ng/ml) which to not impede growth of yeast strains used in this work (see Table S2; results not shown). This observation confirms a previous report [13] and allows for the more general conclusion that adhesion properties of yeast cells are rather sensitive to inhibition of protein synthesis.

Adhesion plays also an important role in cancer metastasis and mammalian eIF4E and eIF4E-BPs have been shown to be involved via the mTOR pathway (for a review, see [33]). Adhesion and invasion require the proper expression of certain mRNAs and we would like to anticipate that beside evident differences between eukaryotic microorganisms and mammalian cells there will be common features in the way how cap-dependent translation is modulated to enhance or repress the expression of certain mRNAs involved in such processes. A careful analysis of the influence of mutants such as those described in this paper on gene expression patterns of haploid and diploid yeast strains will allow to further approach these questions.

## Supporting Information

Figure S1 **Temperature sensitivity of eIF4E mutants.** Serial 1∶10 dilutions of all haploid eIF4E mutants were plated out and incubated on YPD at 30° or 35°C for 2 days, at 37°C for 3 days.
(DOCX)

Figure S2 **NMR structure of yeast eIF4E in complex with m^7^GDP.** Residues in the cap-binding site of eIF4E are displayed. E103, E105, D106 and E107 are marked in red, W104 in yellow and W75 in white, the backbone protein is displayed in yellow (PDB file - 1AP8). m^7^GDP is shown in blue, indicated are the positions of the positively charged 7-methyl imino group and the negatively charged phosphate groups.
(DOCX)

Figure S3 **eIF4F knockouts** Δ**tif3 and** Δ**tif4631 loose adhesion and pseudohyphenation. (A)** Adhesion of haploid Δtif1, Δtif2, Δtif3, Δtif4631 and Δtif4632 deletion mutants in comparison to wt. Plates were incubated at 30° or 35°C for 2 days, then washed under a gentle stream of water. **(B)** Pseudohyphenation of diploid deletion mutants in comparison to wt. Cells were incubated on SLAD_50_ (50 µM ammonium sulphate) plates at 30°C for 2 days; shown is a 200× or 40× magnification of cells. **(C)** ß-Galactosidase activity expressed from *Flo11-LacZ* in haploid eIF4E wt and deletion mutants Δtif1, Δtif2, Δtif3, Δtif4631 and Δtif4632. Expression levels were normalized to LacZ mRNA content which was determined by quantitative RT-PCR. Though normalized LacZ values for Δtif3, Δtif4631 and Δtif4632 are in accordance with the observed haploid and diploid phenotypes, we determined low lacZ values for Δtif1 and Δtif2 which do not correlate well with their phenotype.
(DOCX)

Table S1 **Yeast-2-Hybrid interactions of eIF4E mutants with p20 or Tif4631 peptide (amino acids 391–491).** 2-Hybrid interactions were qualitatively analysed for yeast diploid cells carrying the bait and prey plasmids indicated on plates without histidine (−H) or without adenine (−A): (+++) determines strong, (++) moderate, (+) reduced, (−) no interaction. Interactions were also quantitatively determined as beta-galactosidase (LacZ) Units (duplicate determinations with standard deviation) using cell extracts obtained from diploid cell lines grown at 30°C in SD minimal medium (supplemented with final 20 µg/ml methionine, lysine, histidine, uracil and adenine). Full length p20 or Tif4631 peptide (amino acids 391–491) were cloned as EcoRI/SalI fragments into Yeast-2-Hybrid prey vector pOAD; eIF4E was cloned as EcoRI fragment into the bait vector pOBD2. To obtain eIF4E mutants, site-directed mutagenesis was performed on pOBD2-eIF4E plasmid (oligonucleotide pairs are described in Table S4). Prey and bait yeast strains pJ69-4 were transformed with respective plasmids, crossed and selected on SD minimal medium (supplemented with final 20 µg/ml methionine, lysine, histidine, uracil and adenine).
(DOCX)

Table S2 **Yeast strains used in this work.**
(DOCX)

Table S3 **Plasmids used in this work.**
(DOCX)

Table S4 **Table S4. Oligonucleotides used in this work.** Oligonucleotide pairs used to introduce mutations in yeast eIF4E ORF (Open Reading Frame) and used for quantitative RT-PCR.
(DOCX)
