# Supplementary figures and images for: eIF4E Is an Important Determinant of Adhesion and Pseudohyphal Growth of the Yeast S. cerevisiae
Source: PLoS One. 2012 Nov 30;7(11):e50773. doi: 10.1371/journal.pone.0050773 (PMC3511313; doi:10.1371/journal.pone.0050773)

**Figure S1**


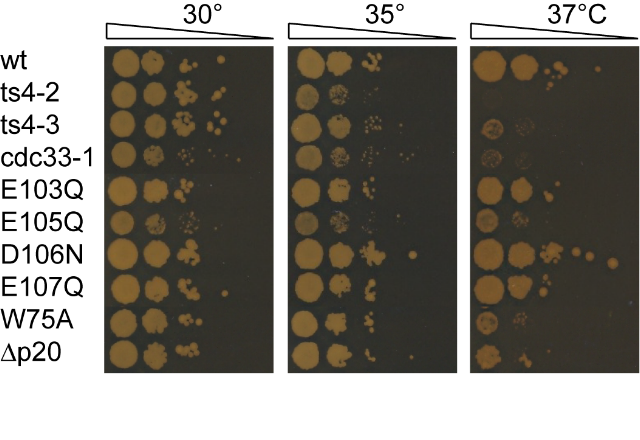

Supplement: Figure S1 — Temperature sensitivity of eIF4E mutants. Serial 1∶10 dilutions of all haploid eIF4E mutants were plated out and incubated on YPD at 30° or 35°C for 2 days, at 37°C for 3 days. (DOCX) [file pone.0050773.s001.docx]

**Figure S2**


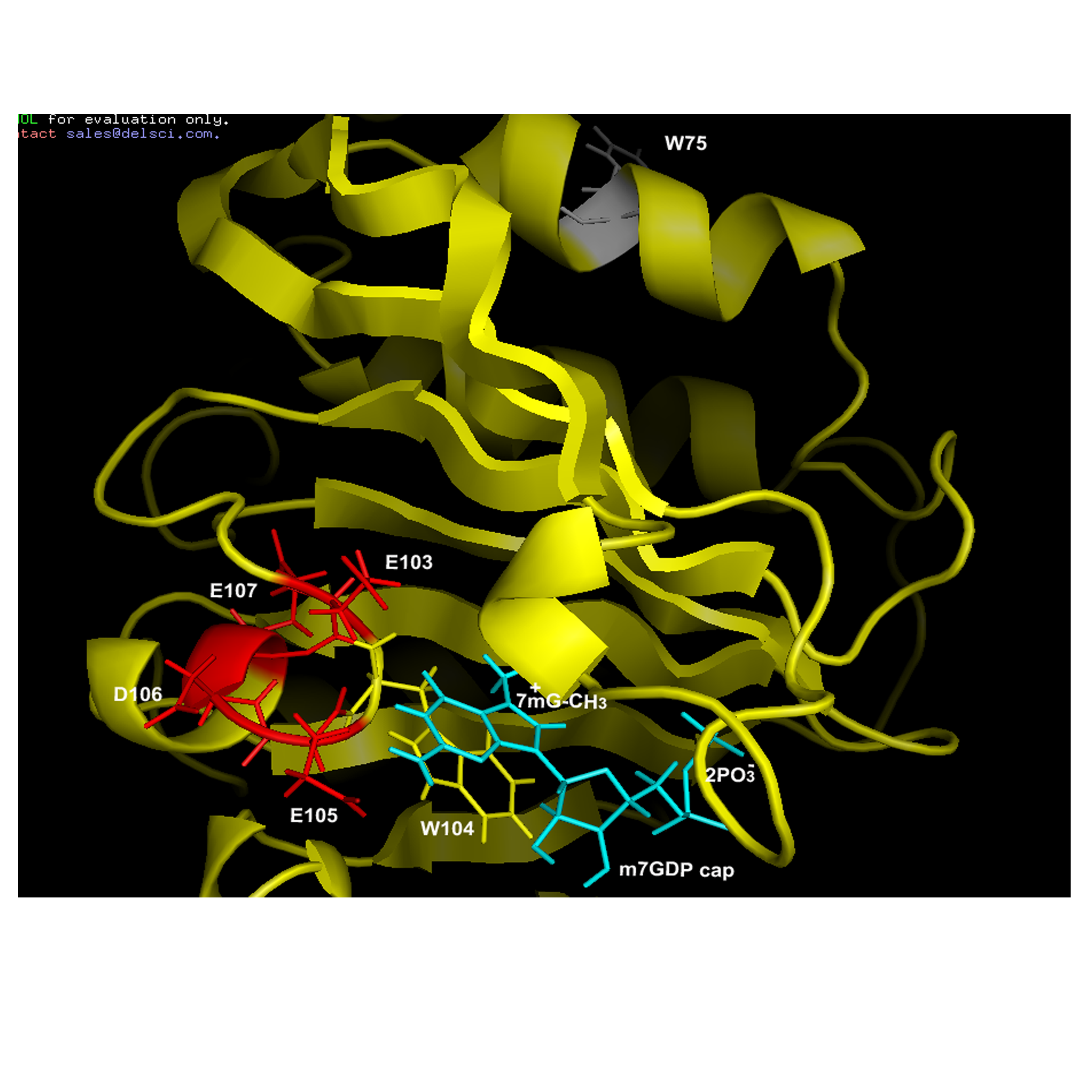

Supplement: Figure S2 — NMR structure of yeast eIF4E in complex with m7GDP. Residues in the cap-binding site of eIF4E are displayed. E103, E105, D106 and E107 are marked in red, W104 in yellow and W75 in white, the backbone protein is displayed in yellow (PDB file - 1AP8). m7GDP is shown in blue, indicated are the positions of the positively charged 7-methyl imino group and the negatively charged phosphate groups. (DOCX) [file pone.0050773.s002.docx]

**Figure S3
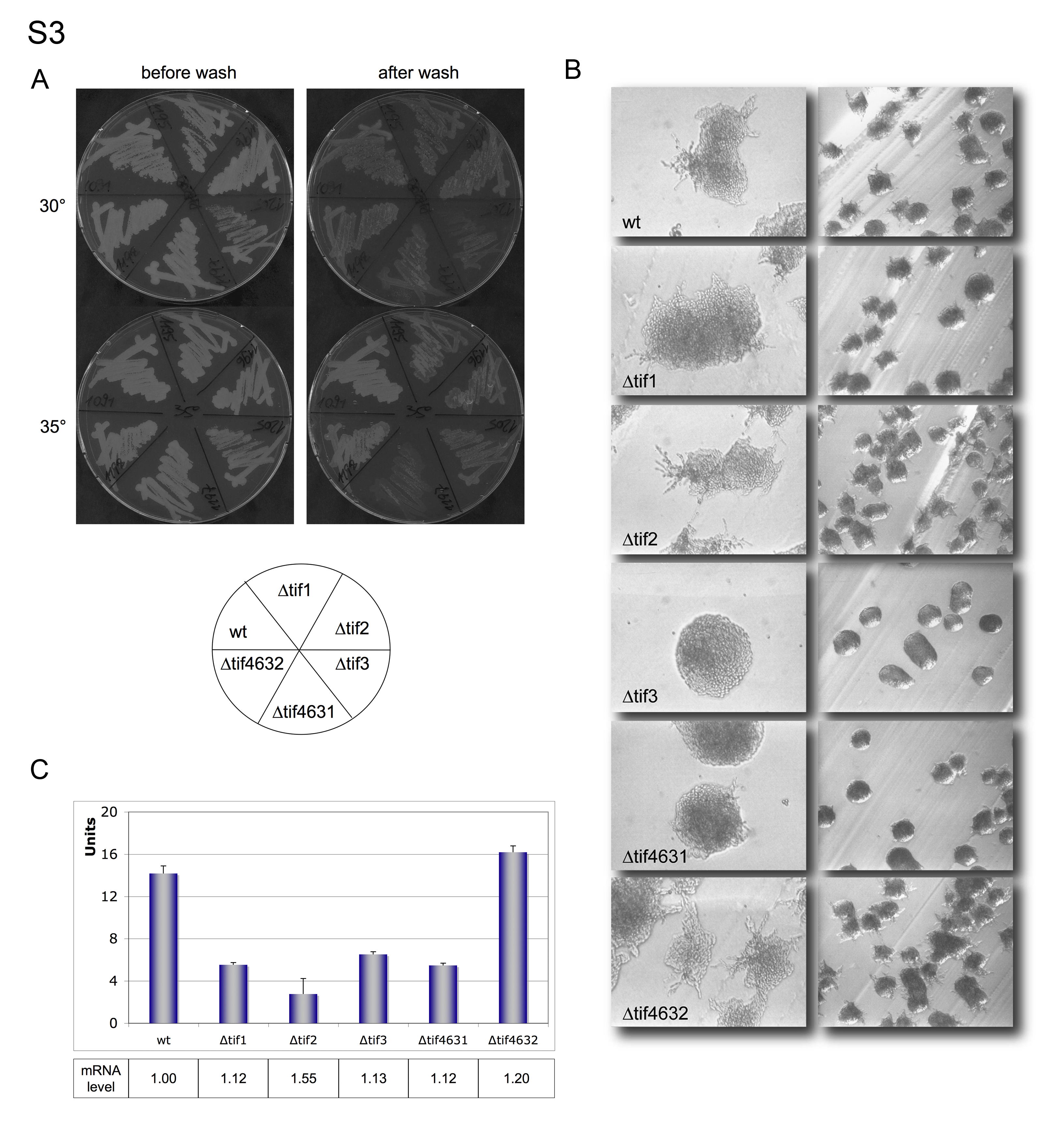
**

Supplement: Figure S3 — eIF4F knockouts Δtif3 and Δtif4631 loose adhesion and pseudohyphenation. (A) Adhesion of haploid Δtif1, Δtif2, Δtif3, Δtif4631 and Δtif4632 deletion mutants in comparison to wt. Plates were incubated at 30° or 35°C for 2 days, then washed under a gentle stream of water. (B) Pseudohyphenation of diploid deletion mutants in comparison to wt. Cells were incubated on SLAD50 (50 µM ammonium sulphate) plates at 30°C for 2 days; shown is a 200× or 40× magnification of cells. (C) ß-Galactosidase activity expressed from Flo11-LacZ in haploid eIF4E wt and deletion mutants Δtif1, Δtif2, Δtif3, Δtif4631 and Δtif4632. Expression levels were normalized to LacZ mRNA content which was determined by quantitative RT-PCR. Though normalized LacZ values for Δtif3, Δtif4631 and Δtif4632 are in accordance with the observed haploid and diploid phenotypes, we determined low lacZ values for Δtif1 and Δtif2 which do not correlate well with their phenotype. (DOCX) [file pone.0050773.s003.docx]
