# Supplementary material for: eIF4E Is an Important Determinant of Adhesion and Pseudohyphal Growth of the Yeast S. cerevisiae
Source: PLoS One. 2012 Nov 30;7(11):e50773. doi: 10.1371/journal.pone.0050773 (PMC3511313; doi:10.1371/journal.pone.0050773)
Supplement: Table S1 — Yeast-2-Hybrid interactions of eIF4E mutants with p20 or Tif4631 peptide (amino acids 391–491). 2-Hybrid interactions were qualitatively analysed for yeast diploid cells carrying the bait and prey plasmids indicated on plates without histidine (−H) or without adenine (−A): (+++) determines strong, (++) moderate, (+) reduced, (−) no interaction. Interactions were also quantitatively determined as beta-galactosidase (LacZ) Units (duplicate determinations with standard deviation) using cell extracts obtained from diploid cell lines grown at 30°C in SD minimal medium (supplemented with final 20 µg/ml methionine, lysine, histidine, uracil and adenine). Full length p20 or Tif4631 peptide (amino acids 391–491) were cloned as EcoRI/SalI fragments into Yeast-2-Hybrid prey vector pOAD; eIF4E was cloned as EcoRI fragment into the bait vector pOBD2. To obtain eIF4E mutants, site-directed mutagenesis was performed on pOBD2-eIF4E plasmid (oligonucleotide pairs are described in Table S4). Prey and bait yeast strains pJ69-4 were transformed with respective plasmids, crossed and selected on SD minimal medium (supplemented with final 20 µg/ml methionine, lysine, histidine, uracil and adenine). (DOCX) [file pone.0050773.s004.docx]

**Table S1**

| pOAD | p20 |  |  |  | Tif4631 (391-491) | |  |  |
| --- | --- | --- | --- | --- | --- | --- | --- | --- |
|  |  |  |  |  |  |  |  |  |
| pOBD | -H | -A | LacZ Units | ± | -H | -A | LacZ Units | ± |
| eIF4E wt | +++ | +++ | 25.6 | 0.3 | +++ | +++ | 14.8 | 0.4 |
| vector | - | - | 2.8 | 0.2 | + | - | 1.7 | 0.0 |
| E103Q | +++ | +++ | 31.6 | 1.6 | +++ | ++ | 14.8 | 0.3 |
| E105Q | +++ | +++ | 39.6 | 2.3 | +++ | ++ | 16.2 | 0.2 |
| D106N | +++ | +++ | 39.3 | 2.2 | +++ | ++ | 12.6 | 0.9 |
| W75A | - | - | 5.7 | 0.0 | ++ | - | 5.5 | 0.1 |
|  |  |  |  |  |  |  |  |  |
