# Supplementary material for: eIF4E Is an Important Determinant of Adhesion and Pseudohyphal Growth of the Yeast S. cerevisiae
Source: PLoS One. 2012 Nov 30;7(11):e50773. doi: 10.1371/journal.pone.0050773 (PMC3511313; doi:10.1371/journal.pone.0050773)
Supplement: Table S2 — Yeast strains used in this work. (DOCX) [file pone.0050773.s005.docx]

**Table S2**

| Name of strain | Genotype and specifications |
| --- | --- |
| RH2585 | *MAT*α, *ura3-52, trp1::hisG, his3::hisG* |
| RH2585 ΔeIF4E::KanX <pVTU-4E> | *MAT*α, eIF4E::KanX, *ura3-52, trp1::hisG, his3::hisG*; essential eIF4E activity provided by <pVTU4E> |
| RH2585 ΔeIF4E::KanX <pCEN16-4E wt/mutation> | *MAT*α, eIF4E::KanX, *ura3-52, trp1::hisG, his3::hisG*; essential eIF4E activity provided by <pCEN16-4E wt/mutation> |
| RH2585 ΔeIF4E::KanX ∆p20::NatR <pCEN16-4E wt/mutation> | *MAT*α, eIF4E::KanX ∆p20::NatR *ura3-52, trp1::hisG, his3::hisG*; essential eIF4E activity provided by <pCEN16-4E wt/mutation> |
| RH2586 ΔeIF4E::KanX <pVTU-4E> | *MATa,* eIF4E::KanX *ura3-52, leu2::hisG, his3::hisG*; essential activity provided by <pVTU4E> |
| RH2586 ΔeIF4E:: NAT^R^ <pVTU-4E> | *MATa,* eIF4E::NAT^R^ *ura3-52, leu2::hisG, his3::hisG*; essential activity provided by <pVTU4E> |
| pJ69-4 MATa | prey strain for Yeast-2-Hybrid selection *MATa, trp1-901 leu2-3, 1 12 ura3-52 his3-200 gal4∆ gal80∆ lys2::GAL1-HIS3 ade2::GAL2-ADE2 met2::GAL7-lacZ* |
| pJ69-4 MATα | bait strain for Yeast-2-Hybrid selection *MATα, trp1-901 leu2-3, 112 ura3-52 his3-200 gal4∆ gal80∆ LYS2::GAL1-HIS3 GAL2-ADE2 met2::GAL7-lacZ* |
