# Supplementary material for: eIF4E Is an Important Determinant of Adhesion and Pseudohyphal Growth of the Yeast S. cerevisiae
Source: PLoS One. 2012 Nov 30;7(11):e50773. doi: 10.1371/journal.pone.0050773 (PMC3511313; doi:10.1371/journal.pone.0050773)
Supplement: Table S3 — Plasmids used in this work. (DOCX) [file pone.0050773.s006.docx]

**Table S3**

| Name of plasmid | Markers and Specifications |
| --- | --- |
| pVTU-eIF4E | URA3; 2µ, ADH1 promotor |
| pCEN16TRP1-eIF4E | TRP1; CEN16 |
| pOAD | LEU2; prey vector for Yeast-2-Hybrid |
| pOBD2 | TRP1; bait vector for Yeast-2-Hybrid |
| YEp355 FLO11::lacZ | URA3; 2µ, FLO11 promotor |
