# Supplementary material for: eIF4E Is an Important Determinant of Adhesion and Pseudohyphal Growth of the Yeast S. cerevisiae
Source: PLoS One. 2012 Nov 30;7(11):e50773. doi: 10.1371/journal.pone.0050773 (PMC3511313; doi:10.1371/journal.pone.0050773)
Supplement: Table S4 — Oligonucleotides used in this work. Oligonucleotide pairs used to introduce mutations in yeast eIF4E ORF (Open Reading Frame) and used for quantitative RT-PCR. (DOCX) [file pone.0050773.s007.docx]

**Table S4**

| mutation/gene | oligonucleotide pair |
| --- | --- |
| D106N | GAC CTG AAT GGG AAA ATG AAG CCA ATG CTA A  TTA GCA TTG GCT TCA TTT TCC CAT TCA GGT C |
| E73K | CCA AAC TGT TGA AAA ATT TTG GGC TAT CAT  ATG ATA GCC CAA AAT TTT TCA ACA GTT TGG |
| E103K | ATG ACG TTA GAC CTA AAT GGG AAG ATG AAG C  GCT TCA TCT TCC CAT TTA GGT CTA ACG TCA T |
| E103Q | ATG ACG TTA GAC CTC AAT GGG AAG ATG AAG C  GCT TCA TCT TCC CAT TGA GGT CTA ACG TCA T |
| E105Q | GTT AGA CCT GAA TGG CAA GAT GAA GCC AAT G  CAT TGG CTT CAT CTT GCC ATT CAG GTC TAA C |
| E107Q | CCT GAA TGG GAA GAT CAA GCC AAT GCT AAA G  CTT TAG CAT TGG CTT GAT CTT CCC ATT CAG G |
| G113D | GCC AAT GCT AAA GGT GAT AAA TGG TCT TTC  GAA AGA CCA TTT ATC ACC TTT AGC ATT GGC |
| G179D | CCA CTA TTG AGA ATT GAT GGT AAA TTC AAG C  GCT TGA ATT TAC CAT CAA TTC TCA ATA GTG G |
| W75A  LacZ  Act1  Fba1 | CTG TTG AAG AAT TTG CGG CTA TCA TTC AAA AT  ATT TTG AAT GAT AGC CGC AAA TTC TTC AAC AC  CCT TGC AGC ACA TCC CCC T  CGC GCC ATT CGC CAT TC  GGA AAT CAC CGC TTT GGC TC  AAC CAC CAA TCC AGA CGG AG  AAC GGT GGT GCT GCT TAC TT  CCG TAA GCT GGA GCA ATG GA |
